# Untargeted and targeted fortified balanced energy-protein (BEP) dietary supplementation during pregnancy and birth outcomes: a cluster-randomised effectiveness trial in rural Bangladesh

**DOI:** 10.1136/bmjgh-2026-023766

**Published:** 2026-06-25

**Authors:** Parul Christian, Hasmot Ali, Eleonor Zavala, Diwakar Mohan, Towfida Jahan Siddiqua, Rezwanul Haque, Hasan M Sujan, Kaniz Ayesha, Lee S F Wu, Brian Dyer, Daniel J Erchick, Andrew L Thorne-Lyman, Kaosar Afsana

**Affiliations:** 1 International Health, Center for Human Nutrition, Johns Hopkins University Bloomberg School of Public Health, Baltimore, Maryland, USA; 2 The JiVitA Project, Rangpur, Bangladesh; 3 BRAC James P Grant School of Public Health, BRAC University, Dhaka, Bangladesh

**Keywords:** Pregnancy, Nutrition, Cluster randomized trial, Global Health

## Abstract

Antenatal balanced energy-protein (BEP) dietary supplementation is a WHO recommendation in undernourished settings, but limited evidence exists on targeting individuals based on nutritional status. We assessed the impact of untargeted and targeted fortified BEP supplementation during pregnancy on birth outcomes, compared with multiple micronutrient supplements (MMS) as control.

In a four-arm, cluster-randomised controlled trial pregnant women received daily: (1) MMS (control); (2) BEP; (3) BEP for those with prepregnancy body mass index (BMI) <18.5, MMS for others and (4) BEP for those with prepregnancy BMI <18.5, MMS for others, with a switch to BEP if inadequate gestational weight gain (IGWG) was identified. Primary outcomes were small-for-gestational age (SGA), birth weight and low birth weight (LBW, <2500 g) assessed within 72 hours of birth. Linear and log-binomial regression models estimated mean differences and relative risks and CIs adjusted for multiple comparisons and design effect.

From October 2022 to November 2024, 3390 pregnant women were enrolled, supplemented and followed in the trial. 2950 women had 2976 live births with 2674 measured within 72 hours of birth. Baseline characteristics did not differ by arm and median adherence was 93%. In the control arm the mean (SD) birth weight was 2763 (383) g, and the incidence of SGA and LBW was 41.4% and 23.4%. Untargeted and targeted BEP supplementation did not differ from the control for primary and secondary outcomes. A higher weight-for-gestational age centile (3.7%, 98.3% CI 0.6% to 6.7%) was seen in the arm targeting low BMI and IGWG. Higher benefits were seen in BEP arms with lower than median adherence compared with the same stratum in the MMS arm and BEP reduced SGA and LBW among anaemic women.

In this high burden Bangladeshi setting, antenatal supplementation with fortified BEP, untargeted or targeted, did not reduce fetal growth restriction and small size at birth when compared with MMS.

**WHAT IS ALREADY KNOWN ON THIS TOPIC**

- Multiple micronutrient supplementation (MMS) versus iron-folic acid and balanced energy-protein (BEP) supplementation during pregnancy have been shown to improve birth weight and reduce incidence of small-for-gestational age births; greater effects of BEP were found in undernourished pregnant women.
- BEP dietary supplementation during pregnancy is recommended by WHO in contexts of high maternal undernutrition.

**WHAT THIS STUDY ADDS**

- This study showed that a fortified daily BEP supplement, containing the same micronutrients as MMS plus calcium in addition to protein and calories, did not reduce incidence of small-for-gestational age or low birth weight births or increase birth weight compared with MMS, provided daily during pregnancy.
- The study also showed a targeted approach for BEP supplementation using risk criteria of low prepregnancy body mass index (BMI) <18.5 kg/m^2^ and inadequate gestational weight gain during pregnancy with the rest getting MMS did not improve birth outcomes compared with MMS alone.
- Targeted BEP reduced low birth weight among women with lower than median supplementation adherence (<93.1%) of BEP and among women with haemoglobin <110 g/L, but not among those with low BMI (<18.5 kg/m^2^) versus the same strata in the MMS group.

**HOW THIS STUDY MIGHT AFFECT RESEARCH, PRACTICE OR POLICY**

- MMS is increasingly gaining momentum in low- and middle-income countries as standard of care during pregnancy and needs to be scaled and integrated as part of antenatal care. BEP supplementation may reduce fetal growth restriction, but further research is needed on optimal doses of calories and protein needed, characterising the dose response relationship and identifying responsive maternal nutritional indicators for more effective targeting.

## Introduction

 Maternal undernutrition and micronutrient deficiencies lead to suboptimal fetal growth contributing to the high burden of small-for-gestational age (SGA), preterm births and subsequent low birth weight (LBW) in low- and middle-income countries (LMICs).^1 2^ To optimise the nutritional well-being of women during pregnancy and to improve birth outcomes, the WHO recommends several evidence-based nutrition interventions. These include (1) iron (30–60 mg) and folic acid (400 µg), (2) balanced energy-protein (BEP) dietary supplements in undernourished contexts, defined as prevalence of low body mass index (BMI <18.5 kg/m^2^) in women of reproductive age of greater than 20% and (3) multiple micronutrient supplements (MMS) containing 15 vitamins and minerals at a single RDA for pregnancy in the context of rigorous research. In addition, nutrition education to increase daily energy and protein intake is recommended to reduce the risk of LBW.^3^

The recommendation for BEP is based on over four decades of research.^4^ ‘Balanced’ protein is defined as food supplements that provide <25% of energy from protein.^5^ A meta-analysis of heterogeneous trials with respect to comparison groups, study size and type and composition of supplements found moderate evidence for a reduction in SGA (7 trials, 4408 women, relative risk (RR): 0.79, 95% CI 0.69 to 0.90) and stillbirths (5 trials, 3408 women, RR: 0.60, 95% CI 0.39 to 0.94) and a mean increase in birth weight of 41 g (95% CI 4.7 to 77.3).^5^ BEP supplementation showed a higher impact on birth weight (67 g) in trials in which most enrolled participants had low prepregnancy or early pregnancy weight compared with trials where participants appeared ‘adequately nourished’ which showed no impact (16 g).^5^ Although antenatal MMS compared with iron-folic acid significantly reduces LBW (by 12%) and SGA (by 8%),^6^ the prevalence of LBW and SGA remains high in many LMICs, especially South Asia, and antenatal BEP supplementation may be needed.^1^

Implementation of the context-specific BEP recommendation has faced programmatic barriers, notably the lack of a standard composition and format appropriate for antenatal use. To address this, an expert consultation resulted in the development of the specifications for a nutritious BEP food supplement designed as a ready-to-use, single daily portion fortified with multiple micronutrients and calcium^7^ and being tested in trials using iron-folic acid as the comparator.^8^,^11^

Given the evidence for MMS as a comprehensive and cost-effective intervention,^12 13^ combined with a global trend of increasing BMI,^14^ and the proposition that a more precise (ie, targeted vs universal) BEP supplementation approach may be safer, more effective and lower cost,^2^ there is a knowledge gap to fill for future design of programmes and delivery of BEP. To address this gap, we undertook a cluster-randomised effectiveness trial in rural Bangladesh. We compared antenatal fortified BEP dietary supplementation to all women versus targeted to high-risk groups (with others receiving MMS) compared with the control of MMS alone as the standard of care. The primary outcomes were SGA, birth weight and LBW, and secondary outcomes were birth anthropometry. We hypothesised that untargeted (universal) BEP supplementation or complementary targeted BEP and MMS would benefit birth outcomes more than MMS alone.

## Methods

The ‘Target-BEP’ trial was conducted by an investigative team at the JiVitA Project site in rural northwestern Bangladesh, the Johns Hopkins Bloomberg School of Public Health, and the BRAC University James P. Grant School of Public Health. The study protocol has been published previously.^15^

### Study design

**Figure 1:**
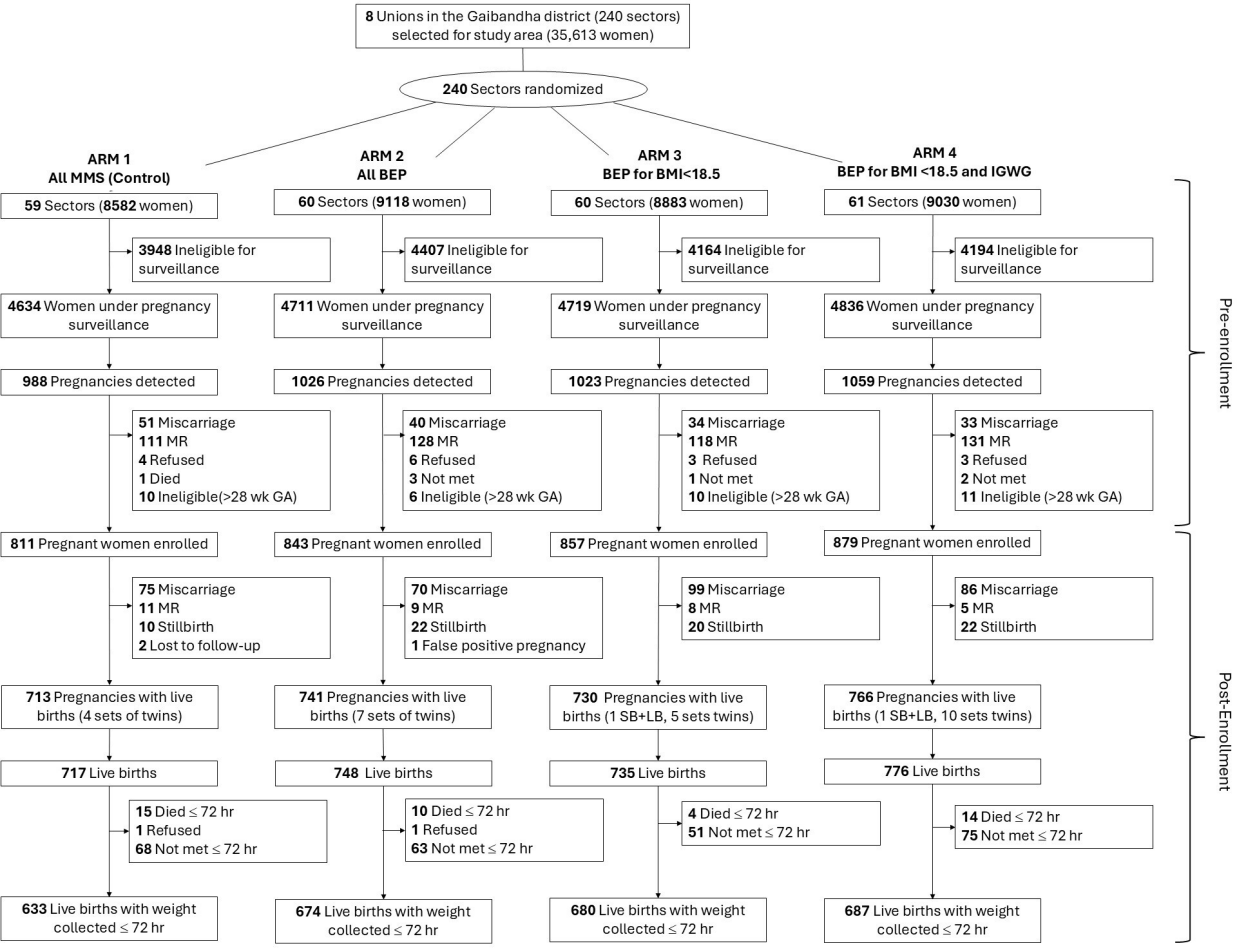
Study flow and participants in the TargetBEP Trial, Bangladesh. BEP, balanced energy-protein; BMI, body mass index; GA, gestational age; IGWG, inadequate gestational weight gain; LB, live birth; MMS, micronutrient supplement; MR, menstrual regulation; SB, stillbirth.

The study was a four-arm, cluster-randomised, open-label effectiveness trial of a daily fortified BEP dietary supplement, given to all pregnant women or targeted to nutritionally vulnerable ones versus MMS (figure 1). Cluster-randomisation was used to manage the logistics of the delivery of the intervention, as well as to reduce cross-contamination and for equity reasons. The study arms were (1) MMS as control, (2) all receiving BEP, (3) BEP to low prepregnancy BMI (<18.5 kg/m²) women and MMS to others and (4) BEP to low prepregnancy BMI women (<18.5 kg/m²) and those who experienced inadequate gestational weight gain (IGWG) and MMS to others. MMS was used as control given prior evidence of its benefit for birth outcomes in the JiVitA area^16^ and globally.^6^

### Study participants and recruitment

The study was conducted in eight Unions of the rural Gaibandha district of Bangladesh. Consenting, newly pregnant married women living in the study area were eligible for enrolment in the trial. A cadre of project community health research workers (CHRWs) enumerated married women (15–35 years), living with their husbands. CHRWs identified new pregnancies by visiting women’s homes every 5 weeks to ascertain their last menstruation date and to administer a pregnancy urine-test if they had missed a period in the past 30 days. Identified pregnant women were visited within 2 weeks by a trained female interviewer (FI) to seek informed consent and to conduct an enrolment interview. In this way we enrolled pregnant women, a large majority in their first trimester, increasing community representativeness in the trial. Enrolment began on 15 October 2022 and ended after 2 years on 11 November 2024, with neonatal follow-ups being completed on 23 April 2025.

### Randomisation and blinding

A ‘sector’ defined in the JiVitA study area comprising 250–300 contiguous households was used as the unit of randomisation. Sectors (n=240) were arranged contiguously and randomised in blocks of 8 by the study statistician using Stata V.15.0 package ‘randomizr’ command for assignment to the four study arms. The residence of the enrolled participant within the sector determined her allocation assignment. Our effectiveness trial was open-label due to the nature of the interventions.^15^

### Study interventions

The interventions included either MMS in the form of the United Nations International Multiple Micronutrient Antenatal Preparation (UNIMMAP) containing 15 vitamins and minerals at a single RDA or a fortified BEP dietary supplement, which provided close to 380 kcals and 14 g of high-quality protein, fortified with the same 15 vitamins and minerals at an RDA plus 500 mg of calcium and 380 mg of phosphorus (online supplemental table 1). The BEP supplement was manufactured by Care Nutrition Ltd (Dhaka, Bangladesh) and was a lipid-based formulation made with rice-lentil or chickpea in three flavours and individually packaged in a daily portion sachet (75 g). The BEP formulation was informed by the specifications and guidance of the expert group.^7^ MMS in a blister pack was manufactured by Renata (Dhaka, Bangladesh).

### Targeting criteria

Our targeting criteria was prepregnancy BMI below 18.5 kg/m^2^ to identify undernourished women and was used in arms 3 and 4 for BEP supplementation. In arm 4, we also targeted women who experienced IGWG. Participants with normal BMI (18.5 to <25) were switched from MMS to BEP using the IGWG cut-off of <3rd percentile of the Intergrowth-21st reference standard,^17^ and for the BMI ≥25 using the Institute of Medicine criteria of <70% of the recommended gestational weight gain.^18^ At the first instance of IGWG in the second trimester, we provided nutrition counselling on increasing daily calories and protein intake as recommended^3^ while continuing MMS. At the subsequent visit identifying IGWG, however, resulted in switching the woman from MMS to BEP for the remainder of her pregnancy. On 18 July 2023, based on the low occurrence of IGWG in the normal BMI women, we deemed the <3rd percentile cut-off as stringent and changed it to <10th percentile. This occurred after 25% of the sample was enrolled and resulted in 3 women being delayed in switching over (mean delay of 7 days). Four women were not switched as they had had an outcome.^15^

### Supplement delivery and adherence assessment

Once a woman reached 12 weeks of gestation, following enrolment, the CHRW visited her home and provided a 40-day supply (10 extra) of her allocated supplement. ANC and nutrition counselling and instructions on the proper use of the supplements were provided to all women in the study by the CHRWs. Subsequently, every 30 days they were revisited to replenish the exact number of consumed supplements, to collect and record empty blister packs and/or sachets and count the days on a calendar on which women had marked their daily use. Two weeks after the in-person visit, women were reached by phone to elicit a 2-weekly history of their supplement consumption and whether the full, two-thirds, half, one-third or none of the BEP was consumed and number of days when supplements were shared with others.

Adherence was calculated as the total number of supplements consumed divided by the number of days between 12 weeks gestation to pregnancy outcome expressed as percent. Supplements consumed was the sum of BEP packets or MMS tablets replenished at monthly visits. For BEP, total amount consumed was adjusted for the woman’s pattern of partial consumption over 2 weeks. For women who switched from MMS to BEP, adherence preswitch and postswitch was calculated using the total tablets/packets consumed for each, divided by the number of days for their respective time periods: 12 weeks gestation to the date of switch and the date of switch to the pregnancy outcome date.

Towards study end (September 2024), a brief stock-out of the BEP supplements due to delays in manufacturing and shipping resulted in 251 participants receiving an alternative supplement scheme, which included an MMS tablet plus an unfortified BEP packet (median duration: 16 days, IQR: 1 day). The scheme matched closely the fortified BEP composition, and adherence during this period was found to be similar to before.

### Outcome measures

All study outcomes were measured by trained research staff with experience in JiVitA. The primary study outcomes were weight measured within 72 hours of birth and used to calculate the incidence of LBW (<2500 g) and SGA defined as weight below the 10th percentile for gestational week using the Intergrowth-21st newborn reference standard.^19^ Gestational age at birth was calculated as days between reported date of last menstrual period (LMP) and date of birth. Gestational age was considered plausible using birth weight for gestational age and sex within ±5 SD using Intergrowth-21st standards (n=2483). For gestational ages not meeting these criteria (n=171), pregnancy surveillance data were reviewed and information from the prior LMPs and date of positive pregnancy tests were used to manually set the LMP (n=65). For the rest, the self-reported gestational age at delivery was used to back calculate the LMP (n=106).

Secondary outcomes included birth length, head and chest circumference also assessed within 72 hours of birth, length-for-age Z-score (LAZ) and weight-for-length Z-score (WLZ) and proportion stunted (<−2 LAZ) and wasted (<−2 WLZ), large-for-gestational age (LGA, >90th percentile of Intergrowth-21st reference standards). Other prespecified but not secondary outcome included weight-for-gestational age centile.

### Data collection and assessments

Our protocol paper^15^ provides details of the data collection methods and assessments. At enrolment, following consent, trained and experienced FIs conducted home-based interviews using digitised forms on tablets to collect household socioeconomic status, previous pregnancy history, 7-day food frequency and 30-day morbidity and assessed weight and mid-upper arm circumference (MUAC), using standard anthropometric techniques. Household food insecurity was ascertained using the Household Food Security Access Scale questions^20^ and haemoglobin (Hb) was measured using capillary blood and a HemoCue device (Hb-301, HemoCue AB, Angelholm, Sweden). Severely anaemic women (Hb <70 g/L; n=5 at enrolment and 4 in late pregnancy) were provided a 60 day supply of iron-folic acid (140 mg and 500 µg) as treatment. Periodic weight was measured by the CHRWs at visits scheduled according to the WHO antenatal care (ANC) contact guidelines. In the third trimester diet, morbidity, anthropometry and Hb assessments were repeated. Using a robust birth notification system, FIs visited the home or facility to conduct delivery interviews and infant anthropometry. Infant weight was measured using a paediatric Tanita scale to the nearest 10 g. Head and chest circumference and MUAC using an insertion tape and length using a length-board were measured in triplicate and the median was used. Maternal weight and MUAC postdelivery were also measured.

### Sample size

Sample size estimate was based on hypothesised RR reductions in primary outcomes of SGA of 20%, LBW of 24% and a mean increase in birth weight of 83 g, SD of 415 g and assuming baseline incidence of SGA of 50% using our previous trial in this area.^16^ Based on the cluster-randomised design and intra-cluster correlation of 0.005 or less and applying a conservative type 1 error of 0.0125 to account for multiple comparisons and with 80% power, we estimated a sample size of 2400 live births. With 30% fetal loss and 6% loss to follow-up, the enrolment requirement was for 3750 pregnancies.^15^ The selected study area comprising 240 sectors, 60 per arm, was estimated to yield the required sample of 10 live births per sector over the enrolment period of 1.5–2 years.

### Statistical analysis

All statistical analyses were prespecified in a statistical analysis plan (online supplemental materials) developed prior to the start of data analysis. Analyses proceeded in several stages, beginning with rigorous data cleaning and preparation, followed by descriptive evaluation of baseline comparability, and then formal analyses of primary and secondary outcomes. Additional analyses addressed adherence, effect modification (subgroup analysis) and potential biases due to missing data.

Participant flow through recruitment, randomisation, follow-up and analysis was documented in a Consolidated Standards of Reporting Trials (CONSORT) diagram (figure 1). Descriptive statistics summarised baseline characteristics by study allocation arm. In keeping with CONSORT recommendations for effectiveness trials, no formal statistical testing was performed for differences in baseline characteristics between groups. Categorical variables (eg, household demographics, reproductive history, morbidities, socioeconomic factors) were summarised as frequencies and percentages, calculated using non-missing denominators. Continuous variables (eg, maternal anthropometry, Hb) were summarised as means with SD or medians with IQRs, depending on their distribution.

The primary analysis population was defined as all live births with anthropometric indicators measured within 72 hours under a modified intention-to-treat framework, in which participants with complete outcome assessment data were analysed according to their randomised allocation regardless of adherence. All tests for statistical significance account for the clustering of the outcome at the ‘sector’ level (unit of randomisation). Continuous outcomes are presented as means with SDs with p values based on analysis of variance. Categorical outcomes are presented as frequencies and percentages, calculated using non-missing denominators with p values based on Scott-Rao χ^2^.^21^

To examine intervention effects, continuous outcomes including birth weight and newborn anthropometry were analysed using linear regression models with generalised estimating equations (GEE) to account for the clustered design of the trial. Effect sizes were expressed as absolute mean differences between the MMS arm and each of the other BEP arms, with corresponding 95% CIs also reported. Binary outcomes, including LBW (<2500 g), SGA (<10 th centile), LGA (>90th centile) were analysed using log-binomial regression with GEE to account for clustering. Results are presented as RRs and 95% CIs. Given the four-arm design, we applied Bonferroni correction to control the total family-wise error rate. For pairwise comparisons on the primary and secondary outcomes, the adjusted alpha threshold for statistical significance was 0.0125 and 98.3% CIs are presented.

Exploratory analyses were conducted to assess effect modification by level of supplement adherence and prespecified maternal and household characteristics measured at enrolment. These included maternal underweight (BMI<18.5 kg/m² and MUAC<23 cm), maternal short stature (<150 cm), adolescent pregnancy (maternal age <20 years), parity (nulliparous vs multiparous), anaemia (Hb <110 g/L), adherence to supplementation (<percent median and supplement count), household food insecurity (any vs none) and infant sex. Effect estimates for all stratified secondary analyses were presented with 95% CIs.

As a sensitivity analysis, adjusted effect estimates were based on regression models incorporating baseline covariates that were correlated with the outcomes. The covariates for adjustment included twin gestation, sex of newborn, maternal BMI, height, age in years and gestational age in weeks at enrolment. As the primary analysis was restricted to infants weighed within 72 hours of birth, this restriction could introduce bias. Secondary analyses were done employing multiple imputations to address missing or delayed weight measurements. For infants weighed between 72 hours and 10 days of life, recalibration of birth weight was performed using the method proposed by Hazel *et al*.^22^ For infants with no recorded weight and other measurements within 10 days, imputations were performed using Multiple Imputation with Chained Equations (MICE) using maternal and pregnancy covariates.^23^ All analyses of the primary and secondary outcomes were replicated using the imputed data.

All analyses were performed using R and packages *survey* and GEE.^24^,^26^ MICE was performed using the *MI* suite of programmes in Stata V.18.

A data safety and monitoring board for this trial was not convened as interventions being provided in pregnancy are those recommended for antenatal use in pregnancy and are considered safe.^3^

### Participant or public involvement

A formative study informed the design of the study intervention and counselling materials. Our hired CHRW cadre are members of the rural community in which the study was conducted. However, participants or the general public were not involved in the conduct of this trial that implemented existing WHO recommended ANC guidelines.^3^

## Results

A community surveillance among 18 900 eligible women of reproductive age identified 4096 incident pregnancies (figure 1). Early fetal losses were the main reasons for attrition, whereas refusals were uncommon and a small number of women were not met. Thus, 3390 pregnant women were enrolled in the trial, started supplementation and were followed through a pregnancy outcome ranging from 811 to 879 pregnancies by arm. Following enrolment, fetal losses, including miscarriages, menstrual regulation and stillbirths, occurred among 406 women. In all, 2950 pregnant women gave birth to at least 1 live birth with a total of 2976 live births and 26 who were twins. Early neonatal deaths or inability to reach participants for an early birth assessment in about 10% newborns resulted in a sample size of infants with anthropometry within 72 hours of 2,674, ranging between 633 and 687 per arm (figure 1).

Baseline characteristics of enrolled pregnant women did not differ by arm (table 1). The mean (SD) age at enrolment was 24.6 (5.6) years and close to 30% were nulliparous. The mean (SD) gestational age at enrolment was 10.3 (3.9) weeks. Women did not differ at enrolment in their height, BMI and MUAC, both prepregnancy and at the time of enrolment in early pregnancy (table 1). The prevalence of BMI <18.5 kg/m^2^ was 19.0% prepregnancy and 17.8% in early pregnancy. Anaemia at enrolment was prevalent among 27.4%. Dietary diversity score and minimum dietary diversity at enrolment did not differ by arm. Women had higher levels of formal education than their husbands. Household food insecurity was low and <5% of households experienced moderate-to-severe food insecurity.

**Table 1:** Baseline and household characteristics of enrolled pregnant women by study arm in the TargetBEP trial, Bangladesh (n=3390)

	All MMS control		All BEP		BEP for BMI <18.5		BEP for BMI <18.5 and IGWG		Overall	
	n=811		n=843		n=857		n=879		n=3390	
	n	%	n	%	n	%	n	%	n	%
Age at enrolment, years, mean (SD)	24.3 (5.5)		24.5 (5.7)		24.9 (5.6)		24.8 (5.6)		24.6 (5.6)	
<19	163	20.1	175	20.8	157	18.3	162	18.4	657	19.4
19–24	321	39.6	295	35.0	303	35.4	321	36.5	1240	36.6
25–29	172	21.2	198	23.5	210	24.5	212	24.1	792	23.4
≥30	155	19.1	175	20.8	187	21.8	184	20.9	701	20.7
Parity										
0	255	31.4	266	31.6	234	27.3	258	29.4	1013	29.9
1–2	500	61.7	510	60.5	544	63.5	531	60.4	2085	61.5
3+	56	6.9	67	7.9	79	9.2	90	10.2	292	8.6
Gestational age at enrolment, weeks, mean (SD)	10.4 (3.9)		10.0 (3.8)		10.4 (4.0)		10.3 (3.7)		10.3 (3.9)	
<13	669	82.5	727	86.2	722	84.2	744	84.6	2862	84.4
13 to <21	118	14.5	99	11.7	103	12	114	13	434	12.8
≥21	24	3.0	17	2.0	32	3.7	21	2.4	94	2.8
MUAC, cm, mean (SD)	26.2 (3.3)		26.2 (3.3)		26.2 (3.3)		26.2 (3.1)		26.2 (3.3)	
<22.5	100	12.3	103	12.2	104	12.1	104	11.8	411	12.1
Height, cm, mean (SD)	150.6 (5.1)		150.6 (5.4)		150.4 (5.3)		150.4 (5.4)		150.5 (5.3)	
<150	363	44.8	367	43.5	406	47.4	398	45.3	1534	45.3
Prepregnancy BMI, kg/m^2^, mean (SD)	21.8 (3.8)		21.7 (3.8)		21.7 (3.6)		21.8 (3.6)		21.7 (3.7)	
Low (<18.5)	153	18.9	168	19.9	169	19.7	154	17.5	644	19.0
Normal (18.5 to <25)	502	61.9	520	61.7	515	60.1	570	64.8	2107	62.2
Overweight (25 to <30)	135	16.6	126	15.0	154	18.0	130	14.8	545	16.1
Obese (≥30)	21	2.6	29	3.4	19	2.2	25	2.8	94	2.8
BMI at enrolment, kg/m^2^, mean (SD)	21.9 (3.8)		21.8 (3.8)		21.8 (3.6)		21.9 (3.7)		21.8 (3.7)	
Low (<18.5)	151	18.6	146	17.3	163	19.0	142	16.2	502	17.8
Normal (18.5 to <25)	491	60.5	539	63.9	522	60.9	578	65.8	2130	62.8
Overweight (25 to <30)	143	17.6	128	15.2	149	17.4	130	14.8	550	16.2
Obese (≥30)	26	3.2	30	3.6	23	2.7	29	3.3	108	3.2
Haemoglobin, g/L, mean (SD)	116.1 (12.0)		116.3 (11.8)		116.1 (11.4)		115.6 (11.7)		116 (11.7)	
Anaemia (Hb <110 g/L)	220	27.5	216	26.1	217	25.6	265	30.6	918	27.4
Dietary diversity score, mean (SD)*	4.5 (1.7)		4.3 (1.6)		4.4 (1.6)		4.4 (1.6)		4.4 (1.6)	
MDD-W†	372	45.9	358	42.5	376	43.9	395	44.9	1501	44.3
Education										
None/informal	36	4.4	32	3.8	42	4.9	42	4.8	152	4.5
Class 1 to class 4	85	10.5	107	12.7	95	11.1	94	10.7	381	11.2
Class 5 to class 9	428	52.8	464	55	468	54.6	492	56	1852	54.6
10 years and above	262	32.3	240	28.5	252	29.4	251	28.6	1005	29.7
Education (husband)										
None/informal	117	14.5	142	16.8	152	17.8	146	16.6	557	16.5
Class 1 to class 4	85	10.5	84	10.0	106	12.4	91	10.4	366	10.8
Class 5 to class 9	320	39.6	335	39.7	315	36.8	350	39.9	1320	39.0
10 years and above	287	35.5	282	33.5	282	33.0	291	33.1	1142	33.7
Living standards index‡										
1st quartile	191	23.6	202	24.0	219	25.6	234	26.6	846	25.0
4th quartile	206	25.4	232	27.5	191	22.3	216	24.6	845	24.9
Drinking water source										
Piped water	3	0.4	4	0.5	2	0.2	5	0.6	14	0.4
Tubewell	808	99.6	839	99.5	855	99.8	874	99.4	3376	99.6
Pit latrines										
Non-flush latrine	794	97.9	825	97.9	842	98.2	867	98.6	3328	98.2
Flush latrine	17	2.1	18	2.1	15	1.8	12	1.4	62	1.8
Household food insecurity§										
None	659	81.4	692	82.2	706	82.5	734	83.6	2791	82.4
Mild	113	14.0	104	12.4	108	12.6	114	13.0	439	13.0
Moderate	29	3.6	36	4.3	22	2.6	21	2.4	108	3.2
Severe	9	1.1	10	1.2	20	2.3	9	1	48	1.4

* Dietary diversity score (food groups consumed ≥4 times/week using a 7-day Food Frequency Questionnaire.

† MDD-W defined as consuming ≥5 food groups.

‡ Index derived using principal components analysis using presence or absence of household assets.

§ Assessed using the Household Food Insecurity Access Scale.^20^

BEP, balanced energy-protein; BMI, body mass index; Hb, haemoglobin; IGWG, inadequate gestational weight gain; MDD-W, minimum dietary diversity, woman; MMS, multiple micronutrient supplement; MUAC, mid upper arm circumference.

Fetal losses in the trial and neonatal mortality did not differ by arm, although a non-significant reduction in neonatal deaths in the untargeted and targeted BEP arms versus the control arm was observed (online supplemental table 2). Online supplemental table 3 shows the newborn condition at birth including type of delivery (35.3% were elective C-sections), place of delivery (48.0% were facility-based) and infant feeding at birth (99% were provided colostrum; 42% were breastfed within 1 hour of birth; 51% were given something other than mother’s milk).

Adherence to supplementation with both MMS and BEP was high in the trial (table 2). In the targeted arms, adherence to both types of supplements was high, ranging from 91% to 100%. Any sharing of the BEP product ranged from 17% to 25% among women who received BEP from the outset but was lower at 11% among those who were switched to BEP. The total number of packets shared over the course of the pregnancy was 5–7 (table 2).

**Table 2:** Adherence to the MMS and BEP supplementation among enrolled pregnancies ending in a live birth with outcome data collected within 72 hours by study arm in the TargetBEP trial, Bangladesh (n=2653)

	All MMS control	All BEP	BEP for BMI <18.5		BEP for BMI <18.5 and IGWG			
	n=630	n=669	n=675		n=680			
Supplement received	MMS	BEP	MMS	BEP	MMS	BEP	Pre-switch (MMS)	Post-switch (BEP)
n, %	630, 100	669, 100	540, 80	135, 20	303, 44.5	123, 18.1	254, 37.4	
GA at first dose, weeks, mean (SD)	13.1 (3.0)	12.9 (2.8)	13.1 (3.0)	13.2 (3.4)	13.0 (3.1)	13.2 (3.3)	12.4 (2.2)	30.2 (4.7)
Eligible supplementation days, mean (SD)*	194.6 (14.6)	193.7 (15.4)	194.8 (14.0)	193.2 (16.7)	190.1 (17.2)	192.3 (13.7)	133.4 (32.8)	63.0 (35.6)
Total tablets/packets consumed†, mean (SD)	171.9 (33.2)	167.8 (44.2)	170.3 (34.2)	162.4 (47.5)	167.0 (36.8)	170.1 (38.6)	115.7 (34.6)	60.9 (35.9)
Percent adherence, mean (SD)	88.0 (15.4)	86.2 (21.3)	87.2 (16.2)	83.8 (23.0)	87.6 (17.9)	87.8 (18.2)	85.6 (16.8)	95.0 (18.0)
Percent adherence, median (IQR)	93.5 (10.6)	94.0 (11.5)	93.4 (11.3)	93.2 (14.3)	94.7 (10.9)	94.5 (10.4)	91.3 (14.8)	100.0 (0.0)
BEP shared, n, %	–	114, 17.0	–	29, 21.5	–	31, 25.2	–	28, 11.0
Total shared BEP packets, mean (SD)	–	6.3 (6.3)	–	6.7 (6.0)	–	6.0 (5.5)	–	5.2 (5.5)

* Eligible days defined from the start of 12 weeks of gestation until birth outcome. Arm 4 preswitch is the number of days between 12 weeks GA to BEP start date. Arm 4 postswitch is the number of days between BEP start date to outcome date.

† Total tablets/packets adjusted for partial consumption, from 12 weeks GA to any outcome.

BEP, balanced energy-protein; BMI, body mass index; GA, gestational age; IGWG, inadequate gestational weight gain; MMS, multiple micronutrient supplement.

Online supplemental table 4 shows the mean (SD) of birth anthropometry and derived indicators and incidence of adverse birth outcomes by study arm.

Table 3 shows the difference between groups relative to the control arm receiving MMS for primary and secondary outcomes at birth among newborns whose birth anthropometry was assessed within 72 hours (n=2674). Mean (SD) birth weight in the control arm was 2763 (383) g and LBW was 23.4% and did not differ between groups. Similarly, SGA was 41.4% in the control group, with RR (98.3% CI) in the BEP arm of 0.92 (0.80 to 1.06), in the BEP for low BMI women arm of 1.04 (0.90 to 1.19), and in the BEP for low BMI and IGWG of 0.91 (0.79 to 1.05). Secondary outcomes of LGA and other birth anthropometric measures were not different by group, except for weight-for-gestational-age centile, which was higher in the arm in which women received BEP if they had low BMI or IGWG (RD: 3.7%, 98.3% CI 0.6% to 6.7%). Adjusted analyses did not change the results and are not presented.

**Table 3:** Birth outcomes and treatment effects by supplementation arm among live births with birth anthropometry assessed within 72 hours in the TargetBEP trial, Bangladesh (n=2674)

	All MMS control n=633	All BEP vs control n=674	BEP for BMI <18.5 vs control n=680	BEP for BMI <18.5 and IGWG vs control n=687
Primary outcomes	Mean (SD) or n (%)	Relative risk/difference (98.3% CI)*		
Birth weight, g	2763 (383)	−2.6 (−49.2 to 44.0)	−24.7 (−71.2 to 21.8)	4.7 (−41.6 to 51.0)
Low birth weight, <2500 g	148 (23.4)	1.02 (0.83 to 1.27)	1.09 (0.89 to 1.35)	0.95 (0.76 to 1.17)
Small for GA (SGA), <10th	262 (41.4)	0.92 (0.80 to 1.06)	1.04 (0.90 to 1.19)	0.91 (0.79 to 1.05)
Secondary outcomes				
Large for GA (LGA), >90th	26 (4.11)	1.11 (0.63 to 1.95)	0.66 (0.35 to 1.26)	1.16 (0.67 to 2.02)
Length, cm†	47.28 (1.92)	−0.10 (−0.33 to 0.13)	−0.08 (−0.31 to 0.16)	−0.11 (−0.34 to 0.13)
Head circumference, cm	33.29 (1.26)	−0.01 (−0.16 to 0.14)	0.02 (−0.14 to 0.17)	−0.01 (−0.16 to 0.15)
Chest circumference, cm	31.84 (1.85)	0 (−0.23 to 0.23)	−0.03 (−0.26 to 0.20)	0.06 (−0.17 to 0.29)
Length-for-age z-score (LAZ)‡	−0.89 (1.10)	0.02 (−0.11 to 0.15)	−0.04 (−0.17 to 0.10)	0.06 (−0.07 to 0.19)
Weight-for-length z-score (WLZ)§	−0.41 (1.02)	0.03 (−0.10 to 0.15)	−0.07 (−0.19 to 0.05)	0.08 (−0.04 to 0.20)
Weight for GA percentile‡	24.3 (26.1)	2.0 (−1.2 to 5.1)	−0.9 (−4.0 to 2.2)	**3.7 (0.6 to 6.8)**

* Relative risk or mean difference from generalised estimating equations with an exchangeable correlation structure, to account for intracluster correlation, for intervention versus control as appropriate.

† Child length missing for n=1. SGA defined as <10th percentile of the IG-21st reference standard; LGA defined as >90th percentile of the IG-21st reference standard.

‡ Mean LAZ and weight for GA percentile at birth calculated using the IG-21st reference standard.

§ WLZ at birth was calculated using the WHO growth standards for term infants and IG-21st reference standard for preterm infants.

BEP, balanced energy-protein; BMI, body mass index; GA, gestational age; IG-21st, Intergrowth-21st; IGWG, inadequate gestational weight gain; MMS, multiple micronutrient supplement.

Sensitivity analysis done to impute birth weight data and models run with imputation with and without adjustment of potential baseline characteristics did not change the results (data not shown). Sensitivity analysis done to examine treatment effects excluding elective C-section deliveries, which are generally done earlier and may have resulted in smaller birth size, did not change the results (data not shown).

Stratified analyses by adherence to supplementation, maternal BMI, anaemia and infant sex are shown in table 4. Higher birth weight was observed in the group that had lower than the median adherence of 93.1% in the all-BEP arm and in the low BMI and IGWG targeted arm versus the control. In the low BMI, IGWG BEP arm, LBW was significantly lower (RR 0.65, 95% CI 0.45 to 0.93) in below the median adherence group compared with the same adherence group in the control. Conversely, in the higher than median adherence group, birth weight was lower in all three BEP arms compared with the MMS arm, although the 95% CI included 0. Similarly, we examined intervention effects by tertiles of the count of supplements (MMS or BEP) and saw a protective effect for SGA and a non-significant 54 g higher birth weight in the BEP arm relative to the control in middle tertile group but not in the highest tertile. In arm 4 (targeted BEP among low BMI and IGWG women), an increase in birth weight of 90 g (95% CI 22 to 158), and reduction in SGA (RR: 0.74, 95% CI 0.58 to 0.93) was observed (table 4). At the same time, the higher than 187 count consumption had lower mean birth weight than in the same count group in the control MMS arm. However, we saw birth weight in the high-count MMS group to be much higher than in the BEP arms (online supplemental figure 1), resulting in the negative impact of BEP on outcomes.

**Table 4:** BEP treatment effects on birth outcomes stratified by adherence, maternal prepregnancy BMI, anaemia and infant sex among live births with birth anthropometry assessed within 72 hours in the TargetBEP trial, Bangladesh (n=2674)

Primary outcomes	All MMS Control		All BEP vs control		BEP for BMI <18.5 vs control		BEP for BMI <18.5 and IGWG vs control	
	Median adherence, %							
	<93.1% n=300	≥93.1% n=333	<93.1% n=296	≥93.1% n=378	<93.1% n=326	≥93.1% n=354	<93.1% n=406	≥93.1% n=281
	Mean (SD) or n (%)		Relative risk/difference (95% CI)*					
Birth weight, g	2730 (376)	2793 (387)	55.3 (−5.3 to 116.0)	−52.6 (−112.6 to 7.3)	8.6 (−52.1 to 69.3)	−55.6 (−112.5 to 1.4)	51.3 (−6.6 to 109.3)	−45.7 (−108.4 to 17.0)
LBW, <2500 g	81 (27.0)	67 (20.1)	0.83 (0.57 to 1.20)	1.29 (0.90 to 1.85)	1.02 (0.72 to 1.45)	1.25 (0.87 to 1.79)	**0.65 (0.46 to 0.93)**	1.41 (0.96 to 2.05)
SGA†,<10th	140 (46.7)	122 (36.6)	0.79 (0.57 to 1.09)	0.98 (0.72 to 1.33)	0.86 (0.63 to 1.17)	1.31 (0.96 to 1.77)	0.79 (0.58 to 1.06)	0.87 (0.62 to 1.21)

	Median adherence, number of tablets/sachets											
	<168 n=224	168–187 n=205	>187 n=204	<168 n=226	168–187 n=205	>187 n=242	<168 n=226	168–187 n=259	>187 n=195	<168 n=231	168–187 n=233	>187 n=223
	Mean (SD) or n (%)			Relative risk/difference (95% CI)*								
Birth weight, g	2675 (374)	2708 (369)	2915 (362)	−18.8 (−92.2 to 54.6)	54.2 (−20.3 to 128.7)	−57.6 (−126.7 to 11.6)	−28.9 (−102.8 to 45.0)	29.1 (−38.3 to 96.4)	**−70.4 (−139.6 to −1.3**)	11.9 (−63.3 to 87.1)	**89.8 (22.0 to 157.7**)	**−96.0 (−168 to −24.1**)
LBW, <2500 g	71 (31.7)	58 (28.3)	19 (19.3)	1.09 (0.84 to 1.42)	0.82 (0.59 to 1.14)	1.57 (0.93 to 2.66)	1.14 (0.88 to 1.48)	0.81 (0.59 to 1.10)	**1.81 (1.06 to 3.07**)	0.87 (0.66 to 1.16)	0.72 (0.52 to 1.01)	**1.91 (1.15 to 3.19**)
SGA†, <10th	80 (35.7)	93 (45.4)	89 (43.6)	0.92 (0.71 to 1.18)	**0.76 (0.60 to 0.97**)	1.06 (0.86 to 1.30)	0.99 (0.77 to 1.27)	0.89 (0.72 to 1.10)	**1.27 (1.03 to 1.55**)	0.81 (0.62 to 1.06)	**0.74 (0.58 to 0.93**)	1.18 (0.97 to 1.45)

	Maternal pre-pregnancy BMI							
	BMI <18.5 n=121	BMI ≥18.5 n=512	BMI <18.5 n=144	BMI ≥18.5 n=530	BMI <18.5 n=135	BMI ≥18.5 n=545	BMI <18.5 n=124	BMI ≥18.5 n=563
	Mean (SD) or n (%)		Relative risk/difference (95% CI)*					
Birth weight, g	2679 (340)	2783 (390)	−53.6 (-135.5 to 28.2)	14.1 (−35.0 to 63.2)	−24.5 (−112.5 to 63.4)	−23.8 (−70.7 to 23.0)	−62.6 (−157.2 to 32.0)	18.4 (−28.6 to 65.3)
LBW, <2500 g	36 (29.8)	112 (21.9)	1.07 (0.74 to 1.54)	1.00 (0.79 to 1.26)	1.21 (0.85 to 1.73)	1.05 (0.84 to 1.31)	1.13 (0.78 to 1.64)	0.90 (0.71 to 1.13)
SGA†, <10th	65 (53.7)	197 (38.5)	1.00 (0.80 to 1.26)	0.88 (0.75 to 1.04)	1.04 (0.82 to 1.30)	1.03 (0.88 to 1.20)	0.77 (0.59 to 1.01)	0.97 (0.83 to 1.13)

	Maternal aenemia							
	Mean (SD) or n %		Relative risk/difference (95% CI)*					
	Hb <110 g/L n=181	Hb ≥110 g/L n=443	Hb <110 g/L n=165	Hb ≥110 g/L n=497	Hb <110 g/L n=181	Hb ≥110 g/L n=495	Hb <110 g/L n=212	Hb ≥110 g/L n=465
Birth weight, g	2704 (400)	2790 (374)	58.1 (−24.0 to 140.2)	−33.2 (−84.0 to 17.6)	−14.9 (−94.0 to 64.3)	−33.3 (−82.5 to 16.0)	**87.4 (9.2 to 165.6**)	−33.8 (−84.8 to 17.3)
LBW, <2500 g	55 (30.4)	90 (20.3)	0.74 (0.51 to 1.06)	1.24 (0.98 to 1.58)	1.07 (0.79 to 1.45)	1.14 (0.89 to 1.45)	**0.62 (0.44 to 0.89**)	1.15 (0.90 to 1.47)
SGA†, <10th	77 (42.5)	180 (40.6)	**0.74 (0.56 to 0.98**)	1.00 (0.86 to 1.17)	1.16 (0.92 to 1.45)	0.99 (0.85 to 1.16)	0.78 (0.61 to 1.01)	0.99 (0.84 to 1.16)

	Infant sex							
	Mean (SD) or n (%)		Relative risk/difference (95% CI)*					
	Female n=313	Male n=320	Female n=327	Male n=347	Female n=322	Male n=358	Female n=354	Male n=333
Birth weight, g	2738 (371)	2788 (393)	−24.9 (−86 to 36.3)	18.2 (−41.5 to 77.9)	−22.2 (−81.9 to 37.4)	−30.6 (−88.9 to 27.7)	−20.4 (−77.5 to 36.7)	33.3 (−29.0 to 95.7)
LBW, <2500 g	76 (24.3)	72 (22.5)	1.20 (0.93 to 1.55)	0.85 (0.63 to 1.15)	1.18 (0.91 to 1.53)	1.03 (0.78 to 1.35)	1.08 (0.83 to 1.40)	0.80 (0.59 to 1.09)
SGA†, <10th	125 (39.9)	137 (42.8)	1.02 (0.84 to 1.23)	0.84 (0.69 to 1.01)	1.07 (0.89 to 1.29)	1.01 (0.85 to 1.20)	0.99 (0.82 to 1.20)	0.83 (0.69 to 1.01)

* Mean difference or relative risk from generalised estimating equations with an exchangeable correlation structure, to account for intracluster correlation, for intervention versus control as appropriate.

† SGA defined as less than the 10th centile of the IG-21st reference standard.

BEP, balanced energy-protein; BMI, body mass index; Hb, haemoglobin; IG-21, intergrowth-21st; IGWG, inadequate gestational weight gain; LBW, low birth weight; MMS, multiple micronutrient supplement.

There was no difference between supplement arms by maternal prepregnancy BMI. Also, universal BEP and targeted to those with low BMI and IGWG reduced SGA and LBW, respectively, in those with anaemia at baseline. A trend for benefit among boys for SGA reduction in the untargeted BEP or those targeted with low BMI or IGWG was observed. Stratified analyses by maternal age, MUAC, height, parity and household food insecurity did not show any effect modification (data not shown).

Dietary diversity and minimum dietary diversity for women in late pregnancy did not differ by supplementation arm (online supplemental table 5).

## Discussion

In our untargeted and targeted effectiveness trial, provision of the WHO recommended daily BEP dietary supplement in a ready-to-eat lipid-based format, fortified with multiple micronutrients and calcium did not impact the primary study outcomes of SGA, birth weight and LBW when compared with a MMS as control provided in a tablet form.

Adherence to supplementation based on tablet/sachet count was high with a median adherence of 93%. However, in both untargeted and targeted BEP arms, lower (not higher) than the median adherence showed benefits for primary outcomes compared with the same group in the MMS control. Low BMI or other maternal nutritional indicators of short stature or low MUAC in the targeted and untargeted arms showed no effect modification. The beneficial effect among anaemic women is notable reflecting this as a high-risk group that could be considered for future targeting. Such an effect modification by maternal anaemia status has been shown previously for MMS versus iron-folic acid,^13^ and it is well recognised that iron-deficiency results in increased iron absorption. There may also be a synergistic effect between protein/energy and nutrient utilisation that resulted in better outcomes. The protective effect for SGA in boys suggests that male and female fetuses may respond differently to maternal nutritional stress and supplementation, an area of ‘sexual dimorphism’ in fetal growth for future research.

There are several possible explanations for the lack of impact of universal BEP supplementation. One is that our trial used the prenatal MMS (UNIMMAP formulation of 15 vitamins and minerals) as the comparison group, shown in 19 efficacy trials to reduce LBW, preterm birth and increase birth weight.^6^ The BEP supplement contained the same amounts of these nutrients but in the lipid-based food form that also contained 500 mg of calcium. One hypothesis is that the bioavailability of micronutrients in the food form could differ from the tablet form, related to the food matrix, especially with added calcium. Interactions between calcium and iron have been posited^27^ and we found that BEP had a significant benefit among anaemic women, suggesting that the calcium–iron interaction, perhaps different in the non-anaemic women, may have masked the beneficial impact of the BEP. Furthermore, adherence to tablet consumption may have been easier and reported more accurately, whereas despite acceptability of the BEP, consuming a full 75 g sachet of the product daily may have been more challenging^3^ and may have been over-reported. In addition, the MMS high adherence arm had a disproportionately better birth weight, rendering the high consumption BEP groups as less impactful, despite the clear dose response seen within groups. Sharing of BEP was also observed in the study.

In the arms in which BEP was targeted to the low BMI group only 18%–20% received BEP continuously throughout pregnancy arm. Also, only 37% received BEP in the late second to third trimester in the IGWG arm and that was in the last 60 days of pregnancy. Although fetal growth is accelerated during the latter part of pregnancy, it is possible that this targeting criteria may result in too short a window to intervene and to reverse fetal growth restriction. Additionally, the daily portions of energy and protein may have been insufficient, and larger quantities may be needed. Also, in the targeted arms the remainder of the women all received MMS resulting in the targeted arms being only marginally different than the control.

Another likely explanation for the null findings could be that the level of calories (380 kcal) and protein (14 g), meeting only under a fifth of the total requirements in pregnancy, may not have been sufficient for reversing the inherent risk conferred by the indicator of undernutrition we used. The current WHO/FAO additional requirements for pregnancy in the second and third trimesters are 285 and 475 kcals, respectively; our supplement met the additional requirements, but macronutrient intake from the home-based diet may have continued to be insufficient in the undernourished women.

Finally, we observed a substantial improvement in the overall nutritional situation among women in our study area, where previously we have seen higher prevalence of low BMI at over 30%.^16^ Similarly, food insecurity has declined and was low in the present study, suggesting adequate availability of macronutrients. Still, SGA incidence has continued to be high at over 40% and the fortified BEP intervention compared with MMS did not result in reducing SGA, our primary outcome of interest.

Recently, BEP trials in Burkina Faso and Ethiopia have also tested fortified BEP compared with iron-folic acid as standard of care^10 11^ and found variable impacts on birth outcomes. Like our trial, the trial in Burkina Faso (called MISAME-III) used a fortified lipid-based BEP supplement that provided 393 kcals and 14.5 g of protein. This intervention did not reduce SGA but increased birth weight by 50.1 g (95% CI 9.1 to 92.0) as well as other anthropometric indicators such as length and gestational age.^11^ The trial in Ethiopia compared an enhanced nutrition package providing a fortified food blend to women with MUAC<23 cm versus iron-folic acid and found no significant impacts on the primary outcomes of birth weight and length, although adherence to BEP was low at 52%.^10^ Similar to arm four in our trial, only 36% of the Ethiopian women were eligible for BEP and consumed on average 61 sachets. However, there was an increase in the coverage for at least four ANC visits in the enhanced nutrition group delivered at health posts, conferring a collateral benefit. In a trial in Burkina Faso (MISAME-II), which provided a lipid-based fortified food supplement with 372 kcals and 14.7 g of protein containing multiple micronutrients compared with the UNIMMAP MMS alone, a significantly higher birth length but not weight was observed.^28^ In a subgroup analysis, higher birth weight of 111 g (p=0.133) and 12 mm length (p=0.005) were observed among underweight women.

Our study had several strengths. Designed to test the effectiveness of BEP supplementation in pregnancy, we followed a rigorous protocol that involved cluster-randomisation of a large number of clusters that resulted in satisfactory balance across a range of characteristics and capture of all community-based pregnancies using a pregnancy surveillance system, increasing the internal validity of the findings. Loss-to-follow-up was limited to early fetal loss or neonatal deaths, with few refusals and out-migrations. Still, some limitations exist. While dietary diversity score did not differ by group, quantitative estimation of the daily food and caloric intakes from home diets was not available to examine replacement in more depth and by adherence. Although we had high reported adherence to supplementation, our monthly in-person visit and phone-based biweekly recall was inadequate, especially to monitor BEP food supplement consumption, generally taken over a few hours during the day and unlike MMS, which was consumed at once as a tablet. Also, reported high adherence for BEP could be due to social desirability even when the full portion was not consumed. Daily direct observation was not feasible and future efforts to improve adherence monitoring are needed. We did not use ultrasonogram-based dating for pregnancies and relied on LMP reporting, although prospectively collected, which may introduce some measurement error. Although we planned secondary between-group and combined intervention group comparisons, these analyses were not undertaken given the null results.

In conclusion, in this rural context in Bangladesh, where MMS has been established to be beneficial for birth outcomes, the addition of supplementary calories, protein and low-dose calcium had no overall additional benefit for fetal growth. Neither did targeted supplementation among low pre-pregnancy BMI women or those who experienced IGWG confer higher benefits although BEP supplementation benefitted those with anaemia requiring further exploration.

Programmatic efforts should continue to focus on MMS scale-up, emphasising the importance of high adherence ensuring daily consumption of supplements and starting early in pregnancy to achieve the most impact. Our trial results may not be generalisable to humanitarian settings or in places where food insecurity is high where a fortified BEP supplement may afford direct benefits for improved birth outcomes and in supporting a healthy pregnancy.

## Supplementary material

10.1136/bmjgh-2026-023766 online supplemental file 1

10.1136/bmjgh-2026-023766 online supplemental file 2

10.1136/bmjgh-2026-023766 online supplemental file 3

## Data Availability

Data are available on reasonable request.
