# Supplementary material for: Untargeted and targeted fortified balanced energy-protein (BEP) dietary supplementation during pregnancy and birth outcomes: a cluster-randomised effectiveness trial in rural Bangladesh
Source: BMJ Glob Health. 2026 Jun 25;11(6):e023766. doi: 10.1136/bmjgh-2026-023766 (PMC13311695; doi:10.1136/bmjgh-2026-023766)

Supplementary Table 1. TargetBEP study supplements and composition.

	Unit	IOM RDA Pregnant Women	Expert consensus specification[7] (per daily serving)	BEP (per daily serving)	UNIMMAP MMS
Energy	kcal		250-500 kcal	382.4	-
Protein	g	71	16 (14-18)	14	-
Fat	g		10-60% of energy	21.8	-
**Micronutrients**					
Vitamin A	µg RE	770	550, 770	770	800
Vitamin D	µg	15	10, 15	15	5
Vitamin E	mg	15	16, 19	16	10
Vitamin K	µg	90 (AI)	72, 90	90	0
Vitamin B9 (Folic acid)	µg	600 (DFE)	400	400	400
Vitamin B1 (Thiamine)	mg	1.4	1.2, 1.4	1.4	1.4
Vitamin B2 (Riboflavin)	mg	1.4	1.3, 1.6	1.4	1.4
Vitamin B3 (Niacin)	mg	18	14, 18	18	18
Vitamin B12	µg	2.6	2.4, 2.8	2.6	2.6
Vitamin B6	mg	1.9	1.7, 2.0	1.9	1.9
Vitamin C	mg	85	100, 120	100	70
Iron	mg	27	22, 27	30	30
Zinc	mg	11	15, 20	15	15
Iodine	µg	220	209, 290	220	150
Copper	µg	1280	1000, 1280	1000	2000
Selenium	µg	60	60, 70	65	65
Calcium	mg	1000	500, 1000	500	0
Phosphorous	mg	700	280, 700	383.6	0

DFE – dietary folate equivalents (or 360 ug folic acid)

Supplementary Table 2. Fetal loss and neonatal mortality among pregnant women enrolled in the trial by study arm in the TargetBEP trial, Bangladesh (n=3390).

	All MMS Control		All BEP		BEP for BMI<18.5		BEP for BMI<18.5 and IGWG		Overall	
	**n**	**%**	**n**	**%**	**n**	**%**	**n**	**%**	**n**	**%**
All Pregnancies	n=811		n=843		n=857		n=879		n=3390	
MR	11	1.4	9	1.1	8	0.93	5	0.57	33	0.97
Spontaneous miscarriage	75	9.3	70	8.3	99	11.6	86	9.8	330	9.7
Live births and stillbirths	n=727		n=770		n=756		n=799		n=3052	
Stillbirths	10	1.4	22	2.9	21	2.8	23	2.9	76	2.5
Perinatal deaths[1]	32	4.4	37	4.8	31	4.1	39	4.9	139	4.5
Live births	n=717		n=748		n=735		n=776		n=2976	
Early neonatal deaths (0-6 days of life)	22	3.07	15	2.01	10	1.36	16	2.06	63	2.12
Neonatal deaths (0- 27 days of life)	25	3.49	17	2.27	12	1.63	19	2.45	73	2.45

MR – menstrual regulation- early termination of pregnancy; IGWG – inadequate gestational weight gain; [1] stillbirths and deaths from 0-6 days of life; None of the p-values using ANOVA was significant

Supplementary Table 3. Newborn condition at birth among pregnant women with any live births or stillbirths in the TargetBEP trial, Bangladesh (n=3024)

	Control All MMS		All BEP		BEP for BMI< 18.5		BEP for BMI<18.5 and IGWG		Overall	
	**n=723**		**n=763**		**n=750**		**n=788**		**n=3024**	
**Birth/delivery condition**	**n**	**%**	**n**	**%**	**n**	**%**	**n**	**%**	**n**	**%**
**Type of delivery**										
Vaginal	435	61.3	473	62.6	469	63.3	490	62.9	1867	62.5
C-section (elective)	260	36.6	265	35.1	255	34.4	273	35	1053	35.3
C-section (indicated)	15	2.1	17	2.3	17	2.3	15	1.9	64	2.1
**Place of delivery**										
Home (own /relative)	329	46.3	363	48.1	379	51.1	364	46.7	1435	48.1
Health worker home	2	0.3	2	0.3	1	0.1	6	0.8	11	0.4
Primary level (community clinic)	16	2.2	11	1.5	13	1.8	23	2.9	63	2.1
Secondary level (District hospital, maternity clinic, NGO or private clinic)	349	49.2	371	49.1	340	45.9	374	47.9	1434	48.0
Tertiary level (Medical college hospital)	5	0.7	5	0.7	4	0.5	9	1.2	23	0.8
Enroute to hospital/ Other	9	1.3	3	0.4	4	0.5	4	0.5	20	0.7
**Condition of the newborn: SB and LB**	**n=727**		**n=770**		**n=756**		**n=799**		**n=3052**	
Malpresentation (vaginal births only)	9	1.26	5	0.66	6	0.8	8	1.02	28	0.93
Baby stuck at delivery	116	16.9	102	14.0	116	16.2	120	16.0	454	15.9
**Condition of the newborn: Live births**	**n=717**		**n=748**		**n=735**		**n=776**		**n=2976**	
It took longer than a minute to breathe/cry	144	20.63	136	18.55	138	19.06	145	19.15	563	19.33
Did not cry or cried weakly at birth	103	14.61	106	14.3	103	14.09	112	14.62	424	14.41
Did not move or moved limbs weakly	82	11.68	94	12.77	89	12.29	106	13.91	371	12.69
Had a part of the body blue at birth	15	2.13	23	3.12	13	1.78	25	3.28	76	2.59
Was provided colostrum at birth	642	99.23	677	99.12	674	99.12	702	99.15	2695	99.15
Breastfed within 1 h of birth	259	40.28	293	43.02	281	41.51	310	43.97	1143	42.24
Something other than mother's milk given since birth	344	52.12	352	49.86	365	51.41	387	52.72	1448	51.53

Supplementary Table 4. Mean (SD) and incidence of birth outcomes by supplementation arm among live births with birth anthropometry assessed within 72 hours in the TargetBEP trial, Bangladesh (n=2674)

	Control All MMS		All BEP		BEP for BMI<18.5		BEP for BMI<18.5 and IGWG		ANOVA / chi2
n	633		674		680		687		
Primary outcomes	n / mean	% / SD	n / mean	% / SD	n / mean	% / SD	n / mean	% / SD	p-value*
Birth weight[2], g, mean (SD)	2763	382.9	2760	408.2	2738	388.2	2767	404.1	0.47
Low birth weight, <2500 g	148	23.4	163	24.2	174	25.6	153	22.3	0.55
Small for Gestational Age, <10%	262	41.4	257	38.1	292	42.9	260	37.8	0.21
Secondary									
Large for Gestational Age, >90%	26	4.1	31	4.6	18	2.6	32	4.7	0.20
Small for Gestational Age, <3%	104	16.4	129	19.1	145	21.3	122	17.8	0.15
Preterm birth, < 37 wk	98	15.5	102	15.1	92	13.5	122	17.8	0.20
GA at birth, wks, mean (SD)	38.9	2.1	38.8	2.2	38.9	2.1	38.7	2.2	0.10
Length, cm, mean (SD)	47.28	1.92	47.18	2.08	47.21	1.98	47.17	2.00	0.81
Head circumference, cm, mean (SD)	33.3	1.3	33.3	1.3	33.3	1.3	33.3	1.3	0.98
Chest circumference, cm, mean (SD)	31.8	1.8	31.8	2.0	31.8	2.00	31.9	2.00	0.89
Length-for-age z-score, mean (SD)	-0.89	1.10	-0.87	1.16	-0.93	1.10	-0.83	1.20	0.57
Weight-for-length z-score, mean (SD)	-0.41	1.02	-0.39	1.04	-0.49	1.03	-0.33	1.01	0.05
Weight-for-age z-score, mean (SD)	-0.95	1.08	-0.89	1.14	-1.01	1.05	-0.82	1.19	0.04
Weight for GA percentile, mean (SD)	0.24	0.26	0.26	0.27	0.23	0.25	0.28	0.28	0.02
Stunted at birth, <-2 LAZ	96	15.2	105	15.6	101	14.8	115	16.7	0.80
Wasted at birth, < -2 WLZ	35	5.5	37	5.5	44	6.5	32	4.7	0.59

*P-values for continuous variables were obtained using adjusted Wald tests of ANOVA from survey design adjusted linear models with clustering at sector level; P-values for categorical variables were obtained using the Rao–Scott adjusted chi-squared test.

GA – gestational age

Mean weight for age z-score (WAZ) and length-for-age z-score (LAZ) at birth and proportion stunted (<-2 Z score) calculated using the IG-21st reference standard and weight-for-length z-score (WLZ) and wasted (<-2 Z-score) calculated using the WHO growth standards for term infants and IG-21st reference standard for preterm infants

Supplementary Table 5. Dietary diversity in late pregnancy by supplementation arm in the TargetBEP trial, Bangladesh

Late pregnancy	All MMS Control	All BEP	BEP for BMI < 18.5	BEP for BMI<18.5 and IGWG	Overall	P-value
n	676	705	707	723	2811	
DDS mean (SD)	5.0 (1.7)	4.9 (1.8)	4.9 (1.7)	4.9 (1.7)	4.9 (1.7)	0.45
MDD-W n (%)	397 (58.7)	402 (57.0)	405 (57.3)	407 (56.3)	1611 (57.3)	0.83

IGWG- inadequate gestational weight gain; DDS – dietary diversity score; MDD-W -minimum dietary diversity – woman

Supplementary Figure 1. Mean birth weight by study arm and tertiles of number of supplements consumed in the TargetBEP trial, Bangladesh.

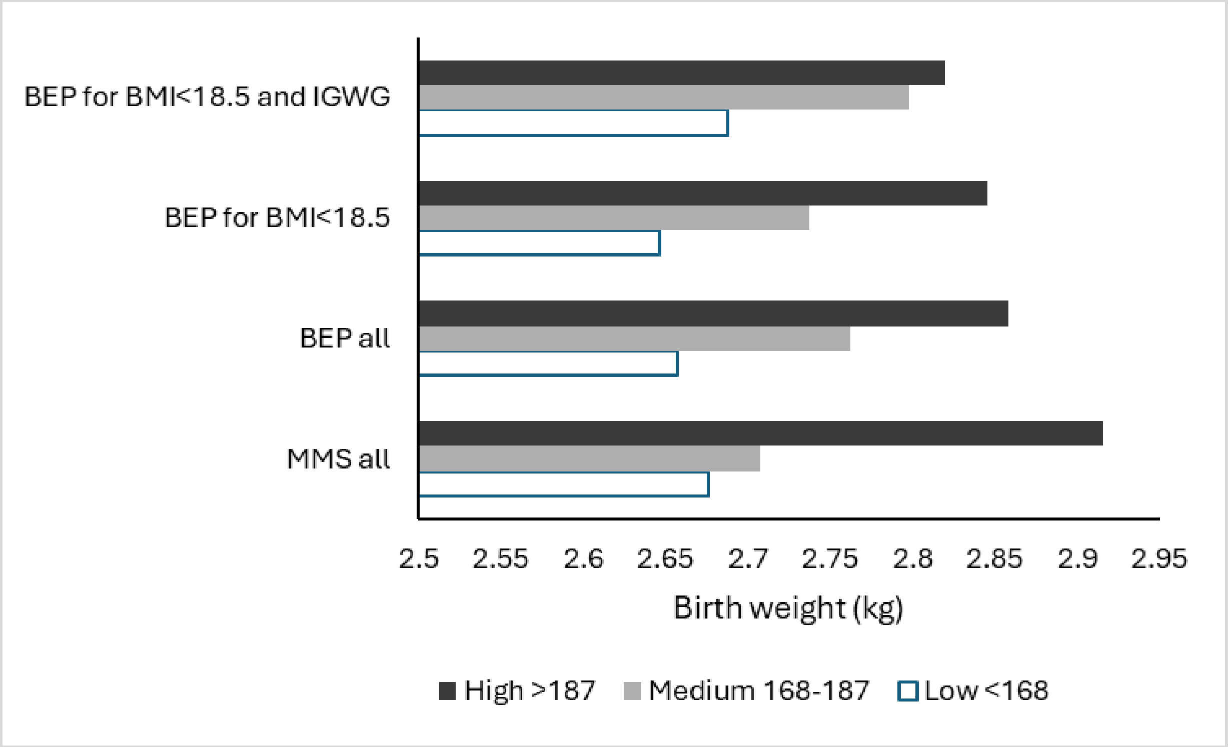

Supplement: online supplemental file 1 [file bmjgh-11-6-s001.pdf]
