# Supplementary material for: Untargeted and targeted fortified balanced energy-protein (BEP) dietary supplementation during pregnancy and birth outcomes: a cluster-randomised effectiveness trial in rural Bangladesh
Source: BMJ Glob Health. 2026 Jun 25;11(6):e023766. doi: 10.1136/bmjgh-2026-023766 (PMC13311695; doi:10.1136/bmjgh-2026-023766)
Supplement: online supplemental file 2 [file bmjgh-11-6-s002.docx]

### BMJ Global Health Author Reflexivity Statement

Adapted from Morton, B., Vercueil, A., Masekela, R., Heinz, E., Reimer, L., Saleh, S., Kalinga, C., Seekles, M., Biccard, B., Chakaya, J., Abimbola, S., Obasi, A. and Oriyo, N. (2022), Consensus statement on measures to promote equitable authorship in the publication of research from international partnerships. Anaesthesia, 77: 264-276. <https://doi.org/10.1111/anae.15597>

| **Study conceptualisation** | |
| --- | --- |
| 1. How does this study address local research and policy priorities? | The prevalence of low birth weight (LBW) and small for gestational age (SGA) remains disproportionately high across many low- and middle-income countries (LMICs). In Bangladesh, maternal undernutrition remains a major public health concern, with approximately 12% of women having a low BMI, ranging regionally from 7.6% to 21.7%. National surveys also indicate a high prevalence of key micronutrient deficiencies. The JiVitA-3 trial, conducted in rural Bangladesh, demonstrated that multiple micronutrient supplementation (MMS) reduced adverse birth outcomes by 10–12% compared to iron-folic acid (IFA). However, increase in birth weight weights were limited (~50g), likely due to high baseline maternal undernutrition (40% low BMI). In such undernourished contexts, especially in South Asia, WHO recommends balanced energy-protein (BEP) supplementation. However, lack of consensus on supplement format and methodology has hindered programmatic progress. An expert consultation recently proposed a ready-to-use BEP formulation. Fortified with micronutrients and low-dose calcium, it provides 250–500 kcal and 14–18g of high-quality protein to meet increased nutritional demands during pregnancy. Although evidence suggests that targeting high-risk (low BMI and inadequate GWG) women may be cost-effective, data remains limited. The Target BEP trial used targeted and untargeted approaches to compare the enhanced BEP product versus standard MMS to assess impacts on increasing birth weight and reducing SGA in rural Bangladesh. |
| 1. How were local researchers involved in study design? | The Target-BEP study represents a 5-year collaboration between researchers from the Johns Hopkins University Bloomberg School of Public Health (JHU) in Baltimore, USA, the James P. Grant School of Public Health at BRAC University (BRAC) in Dhaka, Bangladesh, and the JiVitA Maternal and Child Health and Nutrition Project (JiVitA) in Gaibandha, Bangladesh. From the beginning of the study conceptualization and proposal development, all three organizations were involved. The initiative started with a formative research study, in which BRAC colleagues played a central role in designing the methods, training data collectors, and analysing and interpreting data. These findings directly informed the trial design and implementation. A supplement in the Maternal and Child Nutrition included several papers published as part of this research and several papers were led by BRAC colleagues. Protocol and IRB submissions were drafted by members of all three organizations, with key insights on local considerations provided by JiVitA leadership. |
| **Research management** | |
| 1. How has funding been used to support the local research team(s)? | In the Target-BEP trial, implemented by the JiVitA Project of the Johns Hopkins University INGO employs scientists and organizational leaders from Bangladesh. The director, science, data and field officers supported by administrative and financial staff run the organization /project. The funding support is provided for the Jivita project and is focused on building long-term local research capacity rather than just covering short-term costs. Resources supported a diverse team of Bangladeshi investigators, physicians, and community workers. This ensured that day-to-day operations, from recruitment and consent to field monitoring, were locally led. Additionally, the project provided extensive training in good clinical practice and ethics, while mentorship empowered local researchers to lead data analysis and publications. |
| **Data acquisition and analysis** | |
| 1. How are research staff who conducted data collection acknowledged? | Our field data collectors are acknowledged in the acknowledgements section of the manuscript. Many are co-authors on the paper who have led the science and implementation of the trial. The director of Jivita and Sr Science Officer are also affiliates at Johns Hopkins Bloomberg School of Public Health. |
| 1. How have members of the research partnership been provided with access to study data? | Members of the research team engaged in data analysis have access to the study data. All data is managed by the JiVitA team, and analysis is conducted in collaboration between JiVitA investigators and biostatistician and JHU investigators. |
| 1. How were data used to develop analytical skills within the partnership? | Study data monitoring was conducted by the JiVitA data management team, a team of three individuals who were instrumental to the development of the data collection tools, database management, and dataset preparation and cleaning. The JiVitA biostatistician and data manager were involved in producing analyses and worked hand in hand with the JHU data analysis team to produce study findings. This led to cross-sharing of knowledge and skills related to analytical methods and data visualization. |
| **Data interpretation** | |
| 1. How have research partners collaborated in interpreting study data? | All analytical output was available to senior study members from JiVitA and JHU via a shared drive and discussed on a weekly basis over zoom calls as analyses were ongoing. Additionally, sessions were held at the JiVitA office with the broader local study team to share the results and gather feedback and interpretations from the findings. Meetings with BRAC colleagues to share results further informed interpretation of the findings. |
| **Drafting and revising for intellectual content** | |
| 1. How were research partners supported to develop writing skills? | At all stages of the study initiative, researchers from all three organizations were involved in writing. The study proposal and protocol was led by the JHU PI, with sections written by BRAC, JiVitA, and JHU colleagues. Publications from the formative research study were led by BRAC and JHU study colleagues, where junior researchers from both institutions advanced their writing skills through first author publications. The trial results paper (submitted to BMJ GH) was led by the JHU PI, with written contributions from JiVitA and JHU colleagues. Secondary outcome papers are currently being drafted and led by both JiVitA, BRAC, and JHU researchers at varying levels of seniority and with collaboration across all three organizations. |
| 1. How will research products be shared to address local needs? | Over the course of the study implementation, two stakeholder meetings were held in Dhaka, Bangladesh, with participants from the health and family planning ministries of the Government of Bangladesh, and members of national professional societies, academic and research institutions engaged in maternal and child health. These meetings were co-hosted by JiVitA leadership and BRAC co-investigators. In the first meeting, held on February 28^th^, 2024, results from the formative work were presented, along with the trial objectives and design, followed by discussion with stakeholders on the possible implications of this work. At the second meeting, held on January 5^th^, 2026, trial results were shared with stakeholders and discussions ensued on the implications of the findings. Additionally, local dissemination of study findings will be completed in Q1-Q2 of 2026, at district- and regional-level NGO meetings. |
| **Authorship** | |
| 1. How is the leadership, contribution and ownership of this work by LMIC researchers recognised within the authorship? | The contributors to the research implementation and manuscript drafting are reflected in the manuscript authorship. Five authors from the JiVitA leadership team who were engaged in implementing the study, conducting analyses or interpretation, and drafting/editing the manuscript are included. The senior author is a BRAC university professor and co-investigator who had deep expertise in BEP program implementation in Bangladesh and was engaged in the study design and implementation, and interpretation of the results. |
| 1. How have early career researchers across the partnership been included within the authorship team? | Early career researchers from both JiVitA (2) and JHU (1) are included in the authorship team. These individuals provided important contributions to the study implementation and participated in analysis and/or interpretation of findings, warranting their inclusion in the authorship team. |
| 1. How has gender balance been addressed within the authorship? | We have a gender balance in the authorship, with 6 female authors and 7 male authors, where both the primary and last authors are women. |
| **Training** | |
| 1. How has the project contributed to training of LMIC researchers? | As the study was implemented by JiVitA in Gaibandha, Bangladesh, several JiVitA team members gained experience and training in implementing the study. Two examples are provided, though others also gained valuable research experience. One of JiVitA’s junior research physicians took on more responsibility in this research project, where she largely supported the monitoring of adverse events, shared secondary study findings at an international nutrition conference, and is drafting a manuscript for publication based on those results. Additionally, a junior biostatistician joined the team as part of this project and learned new analytical skills and was exposed to trial implementation, something he had not previously worked on. His writing and problem-solving skills were improved on during this project.  From the BRAC team, two junior researchers were heavily engaged in the qualitative work, improving their research methods and writing skills, and getting exposure to RCT implementation in a community setting. |
| **Infrastructure** | |
| 1. How has the project contributed to improvements in local infrastructure? | The Target-BEP trial significantly improved local infrastructure and institutional capacity. The project strengthened partnerships between BRAC JPGSPH, JiVitA, and Johns Hopkins University, streamlining local ethics processes and ensuring national ownership of data. The project further invested in core research infrastructure by establishing field offices and advanced data systems. This investment included the purchase and maintenance of vehicles essential for transporting field teams and distributing supplements. Such assets will continue to benefit the JiVitA project beyond the trial period. Furthermore, the study supported the local manufacturing sector by sourcing all supplements within Bangladesh. Collectively, this approach represents an equitable research model that leaves behind lasting capacity, physical assets, and leadership. |
| **Governance** | |
| 1. What safeguarding procedures were used to protect local study participants and researchers? | To protect participants and researchers, the study followed rigorous trial procedures. In this effectiveness trial, the interventions are part of evidence-based recommendations of the World Health Organization for antenatal care use, and therefore safety was not a major issue. However, data collection staff were trained to systematically identify and report Serious Adverse Events (SAEs). All SAEs (including death, life-threatening events, hospitalization outside of routine delivery, significant or persistent disability or impairment, and congenital anomalies) were reported from the field to the PI who was responsible in consultation with the investigative team for assessing relatedness to the intervention. Finally, the PI followed the procedures for reporting AEs to the Institutional Review Boards (IRBs). All maternal and infant deaths, and any unanticipated harm, were reported in the main trial publication following CONSORT guidelines.  In addition to monitoring, the trial provide dessential ancillary care to mitigate health risks. Participants reporting severe morbidity or high blood pressure are given medical referrals and travel cost assistance to reach local hospitals. The study also provides direct iron treatment for cases of severe anaemia (< 70 g/L). Finally, to ensure accountability and participant rights, all enrolled women received information cards with direct contact details for the IRBs to report any concerns or perceived harms. |
