# Supplementary material for: Untargeted and targeted fortified balanced energy-protein (BEP) dietary supplementation during pregnancy and birth outcomes: a cluster-randomised effectiveness trial in rural Bangladesh
Source: BMJ Glob Health. 2026 Jun 25;11(6):e023766. doi: 10.1136/bmjgh-2026-023766 (PMC13311695; doi:10.1136/bmjgh-2026-023766)
Supplement: online supplemental file 3 [file bmjgh-11-6-s003.docx]

**TARGETING STRATEGIES FOR BEP SUPPLEMENTATION IN PREGNANCY: TARGET-BEP**

**STATISTICAL ANALYSIS PLAN**

**Version:** 1.0

**Date:** April 19, 2023

**BACKGROUND**

The World Health Organization (WHO) has a contextual recommendation for balanced energy and protein (BEP) dietary supplements for pregnant women to reduce the risk of stillbirth and small-for-gestational age neonates, to be implemented in settings where the prevalence of undernutrition (low BMI) exceeds 20% (1). This recommendation applies to two countries at the national level – India and Bangladesh but applies to many sub-populations in South Asia and sub-Saharan Africa. Bangladesh is categorized as a high-risk country, meeting WHO criteria for implementation of BEP the criteria for implementing the WHO recommendation and has previously emphasized the importance of BEP supplementation for pregnant (and lactating) women as part of their large Bangladesh Integrated Nutrition Program (BINP). The WHO recommendation does not call for screening low BMI women or targeting. Yet, we have recently identified the need for applying an equity lens to women’s nutrition and promoted targeting as a means to safely, enhance impact and cost-effectiveness and reduce overall programmatic cost for antenatal BEP interventions (2).

Pre- or early-pregnancy maternal BMI is a strong predictor of birth outcomes and targeting women with low BMI for supplementation is an effective and safe strategy (2), but data are lacking on the effectiveness of such an approach relative to an untargeted approach. Stratified analysis undertaken as part of the systematic review of BEP interventions has shown a higher effect of 67 g among “undernourished” women compared to 14 g in adequately-nourished women (3), suggesting that undernourished women may benefit somewhat more. Additionally, there may be concerns about increasing BMI among those with a high BMI to begin with. Recently, a review of previous studies in Bangladesh conducted in the context of the BINP and targeting low BMI or low mid-upper arm circumference (MUAC) revealed a variable, albeit a general benefit of supplementation (4). One of the outcomes in these studies has been weight gain in pregnancy, for which there was some evidence of impact with supplementation. As shown in a recent modeled estimate of gestational weight gain in low- and middle-income countries (LMICs) the average weight gain in Africa and South Asia is grossly sub-optimal (5). Gestational weight monitoring in pregnancy including counseling and targeted supplementation based on inadequate weight gain is a strategy that needs to be examined for its effectiveness in improving fetal growth. WHO also recommends counseling for improved dietary intake as an intervention to improve gestational weight gain (GWG). Yet, there is very little evidence of weight monitoring in pregnancy (6), and the potential exists to use the identification of women with insufficient weight gain as an approach to target BEP supplements in food insecure settings.

A fortified balanced-energy protein (BEP) product exists in the form of a lipid-based, energy dense ready-to-eat snack, which will provide 400-500 Kcal energy and 14-16 g of protein, as per the 2017 Expert Consultation on Nutritious Food Supplements for Pregnant and Lactating Women consensus specifications for such a nutritious food product fortified with a total of 19 nutrients including calcium and phosphorous provided at an RDA for pregnant women [(7).](https://gatesopenresearch.org/documents/3-1498)

The proposed effectiveness trial will be conducted with the goal to inform future (BEP) supplementation programs, targeting and delivery models in Bangladesh and the rural Gangetic South Asian setting with a high burden of maternal undernutrition.

**STUDY AIMS**

**Primary aims:**

The primary aim of the study is to evaluate the effect of the BEP supplementation vs. control (multiple micronutrient supplement, MMS) without targeting and with targeting (either by low pre-pregnancy BMI or low pre-pregnancy BMI and inadequate gestational weight gain) on mean birthweight, low birth weight (LBW) (<2,500g), and small-for-gestational age (SGA) (<10^th^ centile using INTERGROWTH 21^st^ standard) as primary outcomes. To do this we are proposing a cluster-randomized, open-labelled effectiveness trial with four arms as shown in Figure 1.

Our main overall aim is:

1. To compare the mean birth weight of infants born to mother randomized across four arms including targeted and untargeted BEP vs. MMS.
2. To compare the prevalence of LBW (<2,500g) of infants born to mother randomized across four arms including targeted and untargeted BEP vs. MMS.
3. To compare the prevalence of SGA (<10^th^ centile using INTERGROWTH 21^st^ standard) of infants born to mother randomized across four arms including targeted and untargeted BEP vs. MMS.

Additionally, to examine the effectiveness of untargeted and targeted BEP supplementation as follows:

1. To compare the mean birth weight, LBW, and SGA of infants born to mothers who were randomized to receive BEP supplementation versus control (MMS) during pregnancy. [arm 2 vs. 1]
2. To compare mean birth weight, LBW, and SGA of infants born to mothers who were randomized to receive BEP supplementation based on low pre-pregnancy BMI versus control (MMS) during pregnancy. [arms 3 vs. 1]
3. To compare mean birth weight, LBW, and SGA of infants born to mothers who were randomized to receive BEP supplementation based on low pre-pregnancy BMI and inadequate gestational weight gain [arms 4 vs. 1]

In addition to the above comparisons, between group differences and 95% confidence intervals will be estimated for both primary and secondary outcomes (shown below), with specific comparisons as described below for the primary outcome of birth weight, SGA and LBW.

1. To compare the mean birth weight, LBW, and SGA of infants born to mothers who received BEP supplementation during pregnancy based on low pre-pregnancy BMI versus untargeted BEP supplementation during pregnancy [arm 3 vs. 2]
2. To compare the mean birth weight, LBW, and SGA of infants born to mothers who received BEP supplementation during pregnancy versus BEP supplementation during pregnancy based on low pre-pregnancy BMI and gestational weight gain. [arm 4 vs. 2]
3. To compare the mean birth weight, LBW, and SGA of infants born to mothers who received BEP supplementation during pregnancy based on pre-pregnancy BMI only versus BEP supplementation during pregnancy based on low pre-pregnancy BMI and gestational weight gain. [arm 4 vs. 3]
4. To compare the *difference* in mean birth weight, LBW, and SGA of infants born to mothers who were randomized to receive BEP without targeting or with targeting relative to the control (MMS). [arm 2 vs 1 VS. arm 3 and 4 vs. 1]

**STUDY DESIGN**

The TARGET-BEP study is a 4-arm, cluster-randomized controlled parallel group superiority trial to assess the effect of targeting strategies for BEP supplementation in pregnancy on birth weight (primary) and other birth outcomes in rural Bangladesh (Figure 1).

**Figure 1: TARGET-BEP Study Design**


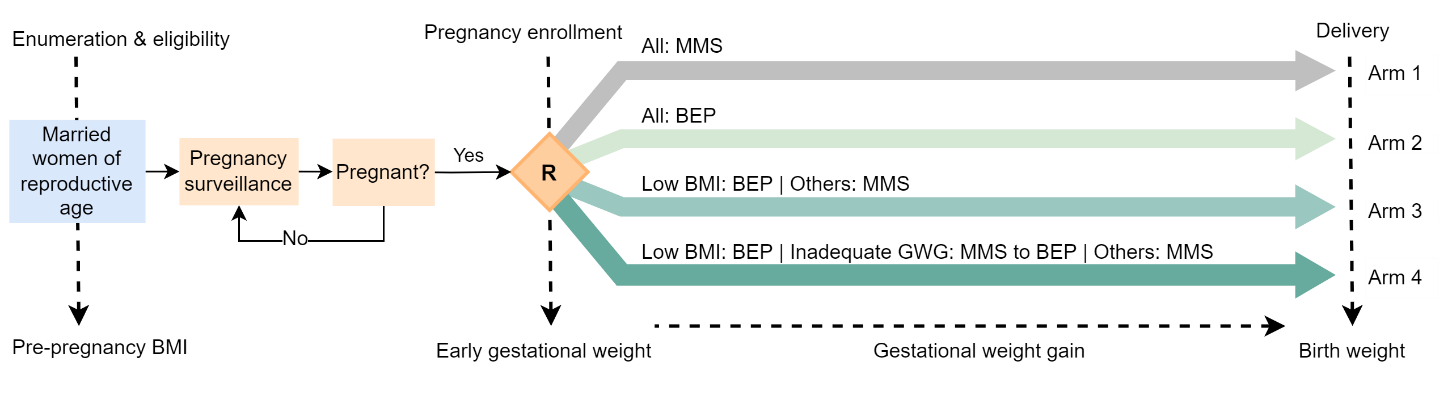


**RANDOMIZATION**

We have randomized at the cluster level using sectors as the unit of randomization, as done in previous JiVitA studies. JiVitA sectors comprise about 250-300 households with women of reproductive age. Cluster-randomization in an effectiveness trial is appropriate because we are testing different targeting approaches for providing BEP, which will allow us to treat women in the same cluster equally in terms of their receipt of the intervention and manage sector workers to avoid contamination between provision of intervention arms.

A total of 240 sectors were randomized to one of the four supplementation arms, with the control arm being provided a daily multiple micronutrient supplement (MMS) as standard of care, given the evidence of benefit to birth outcomes in JiVitA-3 compared to iron-folic acid. We generated these sequences using randomly permuted blocks of size 8 to ensure sequential (i.e., chronological) balance across the groups throughout the enrollment period. We conducted the randomization in Stata 15.0 using the Stata “randomizr” package.

There is no masking of intervention in the trial and, hence, unmasking is not required for analysis.

**SAMPLE SIZE**

Across 240 sectors (60/arm), we will enroll approximately 3,750 pregnancies to obtain 2,400 live births. We assumed a 30% fetal loss and 6% loss to follow-up based on JiVitA-3 data. The sample size at 60 sectors/arm will result in a detectable difference in relative risk of 20% for SGA at 50% prevalence of SGA.

**STUDY OUTCOMES**

**Primary outcomes**

- Infant mean birth weight measured within 72 hours of delivery.
- Proportion low birth weight (LBW) defined as birthweight <2,500g measured within 72 hours of delivery.
- Proportion small-for-gestational-age (SGA) defined as <10th centile of birthweight for gestational age and sex using INTERGROWTH 21st standard measured within 72 hours of delivery.

The secondary outcomes are described in Table 2.

**Table 2: TARGET-BEP study secondary outcomes**

| **Newborn** | **Maternal** |
| --- | --- |
| 1. Mean birth length, head circumference, and chest circumference among live born infants measured within 72 h of delivery. | 1. Mean maternal weight gain and proportion with inadequate weight gain during pregnancy (using the INTERGROWTH 21st GWG reference for normal BMI women and IOM recommendation for low and high BMI women). |
| 2. Mean length-for-age (LAZ) and weight-for-length Z-score (WLZ), and proportion stunted and wasted (< -2 Z) at birth using the WHO Child Growth Study criteria. | 2. Maternal Hb and anemia (Hb < 11g/dl) in the third trimester. |
| 3. Proportion low birth weight (<2,500 g) among live born infants measured within 72 h of delivery. | 3. Maternal postpartum BMI (1 month). |
| 4. Mean gestational age and proportion preterm (<37 weeks) among live born infants. | 4. Maternal dietary intakes in the third trimester (sub-sample). |
| 5. Proportion of SGA (<3rd percentile of the INTERGROWTH 21st standard) among live born infants measured within 72 h of delivery. Mean weight for gestational age/sex centile used for deriving SGA as continuous variable. |  |
| 6. Proportion of LGA (>90th percentile of the INTERGROWTH 21st standard) among live born infants measured within 72 h of delivery. |  |

The key outcome variables of interest are the following:

1. Birth outcome in terms of fetal loss and live birth
2. Number of conceptus - Singleton or multiple gestation
3. Birth weight recorded in grams for live births
4. Gestational age – calculated as difference between date of birth outcome and reported LMP.
5. Weight of pregnant woman at different visits
6. Age at death of liveborn (if died) in the neonatal period

The derived indicators of interest are the following:

1. Proportion of live births that are low birth weight (<2,500g)
2. Proportion of livebirths that are small for gestational age (INTERGROWTH-21st – < 10^th^ and < 3^rd^ centile)
3. Mean birth weight of liveborn
4. Proportion of live births that are preterm (<37 weeks)
5. Mean gestational age at birth of live born
6. Weight for gestational age centile using Intergrowth-21st
7. Mean gestational weight gain per week
8. Proportion of pregnant women with inadequate gestational weight gain (INTERGROWTH-21st – <10^th^ centile and <70% of IOM recommendation)

**Population sets**

**Table 1: TARGET-BEP study population sets**

| Population | Definition |
| --- | --- |
| All enumerated participants | Married women ages 15-35 living with their husbands) |
| All consented | All newly pregnant women and consenting to participate in the trial |
| Full Analysis Set (FAS) | All pregnant women with treatment allocation per randomization status of sector |
| Modified Intent-to-Treat (mITT) set | All pregnant women who provide complete information on birth outcomes. All live births with birth weight measured within 72h of delivery. |
| Per Protocol set | All pregnant women who provide complete information on birth outcomes and have taken BEP with 80% adherence and have no major protocol deviations. |

**STATISTICAL ANALYSIS**

**Data cleaning, manipulation, and preparation**

We will first undertake a preparatory phase including data cleaning, manipulation, and variable transformation, if necessary. This will include preparation of complex variables such as SGA, inadequate GWG, etc. on international standard distributions such as INTERGROWTH 21^st^ and WHO Child Growth Study criteria.

**Analysis of baseline covariates**

We will create CONSORT diagram to show the flow of participants through eligibility, enrollment, randomization, follow-up, and outcome assessment. Next, we will assess baseline comparability by allocation arm before analyzing treatment effects for primary and secondary outcomes. All the variables that are assessed at enrollment; i.e., before BEP or MMS provision, will be considered as baseline characteristics and will be presented by the study arms. Such variables will include household socioeconomic status, household demographic characteristics, reproductive history of the mother, risk factors and morbidities in pregnancy, maternal nutritional status, etc. Following CONSORT guidelines for effectiveness trials, no statistical tests will be conducted comparing baseline characteristics between study arms. Differences in baseline characteristics will be examined by comparing values of means and proportions. The presentation of categorical variables will be done using frequencies and percentages, as appropriate where percentages will be calculated based on the number of participants with non-missing data. Continuous variables will be displayed as mean and SD (Standard Deviation) or median and interquartile range as appropriate, based on their distribution.

**Analysis of primary and secondary outcomes**

The primary outcome analysis will be by modified intention-to-treat and the unit of analysis is singleton live births.

We will analyze continuous outcomes, including the primary outcome of birth weight and other anthropometric measurements, and gestational age, using marginal models and using linear regression with generalized estimating equations to adjust for the trial’s cluster design. We will present absolute mean differences and standardized differences and 95% confidence intervals as measures of impact.

We will analyze binary outcomes, including the primary outcome of LBW and SGA (<10%) and others such as SGA (<3%), using log-binomial regression models with generalized estimating equations to adjust for the trial’s cluster design. We will present relative risk ratios and absolute risk differences and 95% confidence intervals as measures of impact.

If variables are unbalanced at baseline across study arms (defined as more than 2.5% in absolute value), we will assess their associations with rates of the primary and secondary outcomes. If these unbalanced variables are associated with our outcomes, then multivariable analytic approaches will be used to adjust the treatment group differences to control for this imbalance.

**Multiple testing**

To control the family wise error rate, we will use the conservative Bonferroni correction for the type 1 error threshold. Thus, for the four arm comparisons, the alpha threshold will be 0.0125 to reject the null hypothesis of no difference and presenting 98.3% confidence intervals for the primary and secondary outcomes. All stratified analyses and statistical tests for presentation of estimates will be two-sided, at the 5% level of significance.

**Potential stratified analysis**

Stratified analysis will explore for a limited a priori covariates collected at enrollment and known to influence birth outcomes to identify groups that benefit from the intervention. Given that these tests are exploratory in nature, we will not test for significant interaction. We will analyze treatment effects stratified by the covariate(s). The following variables are considered as a priori effect modifiers: Maternal underweight at study inclusion defined as: 1. BMI<18.5 kg/m^2^ (underweight status); 2. MUAC<23 cm; 3. Maternal anemia at study inclusion – Hb concentration<11 g/dL; 4. Maternal short stature: height<145cm; 5. Maternal age < 20; 6. Parity (nulliparous vs. not); 7. Household food insecurity (moderate or severe vs. none, early and later pregnancy). Supplement effect by level of percent adherence (median or tertiles) and supplement dose will also be examined.

**Missing data**

We propose the first analysis to include only weights taken within 72 hours of birth. This is included in our primary outcome definition for mean birthweight, LBW, and SGA. But this will result in some (potentially biased) missing data, which may or may not differ by treatment group. Thus, we propose the following secondary analysis on birth weight. For infants whose weight was taken >72 hours of birth to 10 days of birth, we will multiply imputate birthweights using a model based on longitudinal weight data taken in the Nepal Newborn Washing Study (Tielsch, et al.). The longitudinal data were collected on 457 babies with weights measured within 6 hours of birth and then daily for 10 days thereafter (Hazel, et al). For infants whose weight was taken <72 hours, the time of measurement and the weight was used as well as other covariates (maternal age and education, parity, infant sex, neonatal death, gestational age) to impute weight at time zero. For infants missing weight altogether (or where weight was measured after 10 days), the birthweight will be multiply imputed using these predictors without weight and time of weight measurement. This will allow for a complete set of birthweights for the entire set of liveborn babies in the trial.
